# Vasorin-containing small extracellular vesicles retard intervertebral disc degeneration utilizing an injectable thermoresponsive delivery system

**DOI:** 10.1186/s12951-022-01624-1

**Published:** 2022-09-19

**Authors:** Zhiwei Liao, Wencan Ke, Hui Liu, Bide Tong, Kun Wang, Xiaobo Feng, Wenbin Hua, Bingjin Wang, Yu Song, Rongjin Luo, Huaizhen Liang, Weifeng Zhang, Kangcheng Zhao, Shuai Li, Cao Yang

**Affiliations:** Department of Orthopaedics, Union Hospital, Tongji Medical College, Huazhong University of Science and Technology, Wuhan, 430022 China

**Keywords:** Intervertebral disc degeneration, Nucleus pulposus, Extracellular vesicles, Decellularized extracellular matrix, Thermoresponsive hydrogel

## Abstract

Intervertebral disc degeneration (IDD) is the pathological reason of back pain and the therapeutic approaches are still unsatisfactory. Recently, mesenchymal stem cell-derived small extracellular vesicles (EVs) have emerged as the novel regenerative method for IDD. In this study, we intensively investigated the therapeutic mechanism of small EVs, and found that vasorin protein enriched in EVs promoted the proliferation and extracellular matrix anabolism of nucleus pulposus cells via the Notch1 signaling pathway. Then, we fabricated a thermoresponsive gel which composed of Pluronic F127 and decellularized extracellular matrix (FEC) for the delivery and sustained release of EVs. Besides, ex vivo and in vivo results showed that EVs embedded in FEC (EVs@FEC) ameliorate the disc degeneration efficiently and achieve better therapeutic effects than one-off EVs delivery. Collectively, these findings deepen the understanding of EVs mechanism in treating intervertebral disc degeneration, and also illustrate the promising capacity of sustained EVs release system for intervertebral disc regeneration.

**Supplementary Information:**

The online version contains supplementary material available at 10.1186/s12951-022-01624-1.

## Introduction

Intervertebral disc degeneration (IDD) is considered as a common degenerative musculoskeletal disease and brings heavy healthcare burden to society [1]. Intervertebral disc (IVD), composed of the inner nucleus pulposus (NP) and surrounded by the annulus fibrosus (AF), is an avascular organ that has limited self-healing capacity [2]. During the progression of IDD, NP tissues are diminished gradually, companied with or without the rupture of AF tissues. One of the important features of degenerated NP tissues is the imbalance of extracellular matrix metabolism and decline of resident cells [3]. Degeneration of NP tissues plays a critical role in IDD, resulting in discogenic pain [3]. Besides, current treatments of IDD, including nonpharmacological or pharmacological approaches, could achieve pain-relief effects, valid for a period of time [4]. Surgical procedures, such as discectomy and fusion, remove disc tissues and reconstruct with external fixation, while cannot preserve the structure and function of IVD and may lead to postoperative complications and secondary injuries [4, 5]. Therefore, new approaches to enhance the self-regenerative capacity of degenerated IVD and repair the damaged disc tissues are desperately required for IDD therapy.

Mesenchymal stem cells (MSC)-based tissue regeneration is a promising therapeutic approach in musculoskeletal degenerative diseases [6, 7]. MSCs are considered as hypo-immunogenic and immune-evasive cells, which serve as a potential treatment in bone or cartilage repair [7]. Accumulating evidence declares that MSCs exert the therapeutic benefit mainly via paracrine mechanisms [8]. Extracellular vesicles (EVs) are important bioactive components of MSCs paracrine, promoting the self-healing capacity of musculoskeletal tissues [9, 10]. Compared with MSCs therapy, EVs as a cell-free therapy could be isolated from cells sustainably, and are convenient in reserve and transport [11]. Our previous studies have indicated that MSC-derived EVs could protect NP cells against cell apoptosis or pyroptosis, or rejuvenate the senescent NP cells via the delivery of functional proteins [12–14]. Generally, MSC-derived EVs could serve as a promising treatment for IDD, while the delivery methods of EVs still need to be improved. Some studies suggest that EVs administered by local injection are rapidly cleared from the sites, resulting in the repeated injections in practice [11]. To guarantee the therapeutic expectation of EVs, an effective delivery approach of EVs needs to be further investigated.

Among these EVs, small EVs are nanovesicles with a diameter of 30–150 nm, which are abundant in proteins, lipids and nucleic acids [15]. There is mounting interest in diverse cargoes of EVs, which mediate cell-to-cell communication and alter the phenotype of recipient cells [16]. EVs appear to be involved in treating degenerative diseases and show great potentials in tissue maintenance and repair [17]. Current studies mainly focus on the miRNAs or protein cargoes in EVs, which mediate the phenotypic changes of recipient cells. Once engulfed by recipient cells, EVs release diverse cargoes that induce the cellular signaling transduction [18]. We found that a transmembrane protein, Vasorin (VASN), is abundant in MSC-EVs based on our previous proteomics analysis [13]. Several studies showed that Vasorin has a close relationship with cell migration and proliferation of vascular muscle cells [19–21]. However, the role of vasorin in NP cells remains unclear. Considering the various molecules of EVs with their diverse effects, functional cargoes in small EVs that mediate the regenerative effects in IDD still need more validated researches.

Biomaterials serve as optimized approaches for EVs application in IDD, that offer delivery platforms to guarantee the sustained release of EVs, resulting in an increased EVs retention rate [3, 22]. For IVD tissue regeneration, thermoresponsive hydrogel is an ideal biomaterial for EVs delivery, owing to its characteristics, such as high moisture content, biocompatibility and degradability [23–25]. Recently, decellularized extracellular matrix (dECM) attracts increasing interests in tissue regeneration engineering, which could mimic intrinsic tissue microenvironment and transduce various endogenous bioactive signals [26]. The application of dECM enhances the biocompatibility and cell affinity of synthetic biomaterials for EVs delivery, which could optimize the therapeutic effects of EVs [27]. Here, we fabricated a composite hydrogel based on thermoresponsive Pluronic F127 (F127) gel and human NP tissues-derived dECM, which serves as the delivery approach to realize the sustained released of EVs in IDD regenerative therapy.

**Fig. 1:**
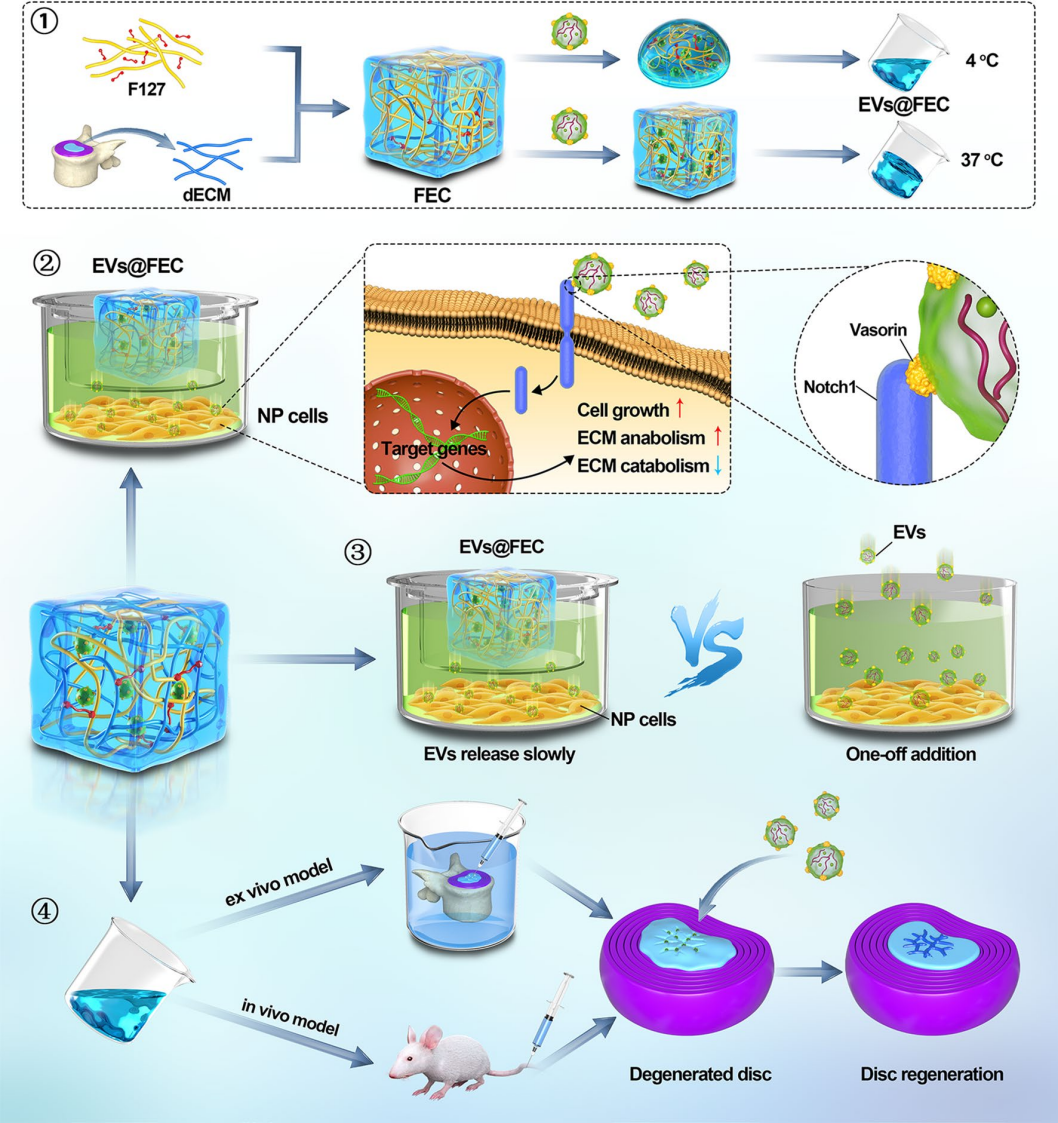
Diagrammatic sketch of hydrogel fabrication and its utilization in intervertebral disc regeneration. (1) Workflow of hydrogel fabrication. (2) Mechanism scheme of EVs-mediated effects on NP cells. (3) Sustained EVs release in FEC hydrogel. (4) Application of EVs@FEC in ex vivo and in vivo disc degeneration model. FEC, composite hydrogel based on Pluronic F127 and decellularized NP tissues (dECM); EVs, extracellular vesicles; EVs@FEC, EVs encapsulated into FEC hydrogel; NP cells, nucleus pulposus cells

In this study, we found that MSC-derived EVs could promote the viability of NP cells and maintain the metabolic balance of extracellular matrix (ECM) via the Notch pathway, which is mainly mediated by EVs protein Vasorin. Then, we developed an injectable thermoresponsive hydrogel as an EVs carrier that displayed an effect of long-term EVs release (Fig. 1). To fabricate the mixed hydrogel, we processed the decellularized NP tissues into gelatinous solution and then was complexed with the F127, a nonionic triblock copolymer with temperature responsive property. Moreover, the regenerative effect of long-term EVs released in the thermoresponsive hydrogel on NP cells was investigated based on the ex vivo and in vivo disc degeneration model. In summary, our research aims to reveal the sustained EVs release effect of thermoresponsive hydrogel and provides an advantageous approach for intervertebral disc regeneration.

## Methods and materials

### Cell isolation and culture

The human primary NP cells were isolated from patients who underwent surgery for disc excision. The experimental design and protocols were endorsed by the Ethics Committee of Tongji Medical College, Huazhong University of Science and Technology. Informed consent was obtained from all involved patients. Briefly, the NP tissues were collected and digested with 0.2% collagenase II for 4 h. After washed and centrifuged, the precipitation was resuspended and cultured in Dulbecco’s Modified Eagle Medium/Nutrient Mixture F-12 (DMEM/F-12) containing 15% fetal bovine serum (HYcezmbio, Wuhan, China). For MSCs isolation, human bone marrow specimens were obtained from the iliac crests of volunteer donors. Human MSCs were then isolated from bone marrow by density gradient centrifugation and cultured in DMEM/F-12 containing 15% fetal bovine serum. The culture medium was replaced once every three days.

### Isolation and identification of EVs

For small EVs isolation, human MSCs were cultured in DMEM/F-12 containing 15% EVs-depleted fetal bovine serum (VivaCell, Shanghai, China) for 24 h. The medium was collected and centrifuged at first 500 × *g* for 10 min, then 2000 × *g* for 30 min, and 10,000 × *g* for 1 h. After filtered through a 0.22-μm filter (Millipore, USA), the remaining supernatant was centrifuged by ultracentrifugation at 110,000 × *g* (Beckman Type 70 Ti, USA) for 70 min twice. The pellet was suspended in PBS for the further experiments. The protein concentration of EVs was evaluated by BCA assay (Beyotime, China). For morphology analysis, the EVs were fixed with 2.5% gluteraldehyde for 30 min and placed on copper grids. Images were captured by transmission electron microscopy (TEM, FEI Tecnai G20 TWIN, USA). The number and size of EVs were assessed by nanoparticle tracking analysis (NTA) using NANOSIGHT NS300 system (Malvern, UK).

### Western blot assay

The cells were lysed in RIPA solution (Beyotime, China) with a protease inhibitor PMSF (Solarbio, China). The isolated proteins were separated by sodium dodecyl sulfate polyacrylamide gel electrophoresis (SDS-PAGE) and transferred onto a PVDF membrane (Millipore, USA). The membrane was washed in 5% milk for 1 h and then incubated with a primary antibody overnight. The antibodies including anti-COL2A1 (28459-1-AP), anti-ACAN (13880-1-AP), anti-MMP3 (17873-1-AP), anti-MMP13 (18165-1-AP), anti-Alix (12422-1-AP), anti-CD63 (25682-1-AP), anti-Notch1 (20687-1-AP), anti-Hey1 (19929-1-AP), and anti-Hey2 (10597-1-AP) were purchased from Proteintech company (Wuhan, China). The antibodies including anti-Vasorin (ab156868), anti-Calnexin (ab133615), and anti-GAPDH (ab8245) were purchased from Abcam company (Cambridge, UK). After incubated with horseradish peroxidase (HRP)-conjugated secondary antibodies, the bands were visualized using an ECL Reagent (Affinity Biosciences, USA).

### Cell proliferation and migration assay

Cell proliferation was assessed using a Cell Counting Kit-8 (GlpBio, USA) according to the manufactural protocol. After NP cells were conducted with the experimental treatments, the CCK-8 solution was added to each well and incubated for 4 h. The samples were measured at 450 nm absorbance using a spectrophotometer (BioTek, USA). Cell migration was evaluated by wound healing assay. NP cells were seeded in 6-well plate and a linear thin scratch was made in the plate. Then the medium was replaced and the images were captured by a microscope (Olympus, USA). The wounded area was measured by ImageJ 1.52a (National Institutes of Health, USA) and the percentage of wounded area was calculated according to the formula: Wounded area filled (%) = (W_b_—W_m_) / (W_b_) × 100%. W_b_ was the width at the beginning and W_m_ was the width at the measured time.

### Quantitative real‐time polymerase chain reaction

NP cells were treated with TRIzol (Invitrogen, CA, USA) RNA isolation reagent. The RNAs were then purified by chloroform, and reverse-transcribed and amplified by quantitative real‐time polymerase chain reaction (qRT‐PCR) according to the previous protocol. The primers used were listed in (Additional file 1: Table S1). GAPDH as an internal control was used for normalization. All experiments were conducted at least in triplicate.

### RNA interfering

Interfering of VASN or NOTCH1 was realized by small interfering RNA (siRNA). The targeted siRNAs (si-VASN) and scrambled siRNA (si-scr) were synthesized by GENERAL BIOL (Anhui, China). The sequences used were listed in (Additional file 1: Table S2). The siRNA interfering was conducted utilizing a transfection reagent (MCE, Shanghai, China) according to the manufactural protocol. The interfering efficacy was measured by quantitative real-time polymerase chain reaction (PCR) at 24 h after transfection.

### Immunoprecipitation

NP cells were treated with PBS or EVs and the cell lysates were collected. The samples were treated with 50 mM Tris–HCl, 150 mM NaCl, 1 mM EDTA, 1% NP-40 with protease inhibitor cocktail (Beyotime, China). The sample (500 μg) was incubated with anti-Notch1 antibody and Protein A/G magnetic beads (MCE, Shanghai, China) overnight, and other remaining samples were used as in-put. After the mixture was separated by magnetic adsorption and washed with PBS twice, the immunoprecipitates were collected and conducted with western blot assays.

### Immunofluorescence analysis

The cells were fixed with 4% paraformaldehyde (15 min), permeabilized with 0.2% Triton X-100 (30 min), and then blocked with 2% goat serum albumin (1 h). The cells were incubated with a primary antibody overnight including anti-Notch1 (10,062–2-AP, Proteintech, China) and anti-Vasorin (227,526, R&D Systems, USA), and then with fluorescent-conjugated secondary antibodies for 1 h in the next day. Images were obtained under a microscope (Olympus, USA) in random fields and the accordingly MFI was analyzed by ImageJ 1.52a (National Institutes of Health, USA).

### Hydrogel preparation and characterization

To obtain decellularized ECM (dECM), the isolated NP tissues were washed with PBS twice, and then treated with 0.25% Triton-X 100 and NH_4_OH (20 mM) for 5 min. Combined with a treatment of DNase I (50 U/mL) and RNase A (100 μL/mL) for 2 h, the nucleic acids were removed from the ECM. The remaining dECM were washed with PBS three times, and lyophilized for further use. The FEC hydrogel solution was prepared by dissolving 15 wt% Pluronic F127 and 0.1 wt% or 1 wt% dECM in deionized water. The mixed hydrogel was placed in vacuum overnight to remove bubbles. For the thermoresponsive ability detection, 1 mL mixed hydrogel was added into a vial and incubated at 4, 25, and 37 °C. Then tilt the vials and observe the changes of the gel surface. For the surface morphology analysis, the hydrogel samples were lyophilized for 48 h and then cut into pieces. The samples were coated by gold with a thickness of approximately 10–20 nm and visualized by a scanning electron microscope (SEM, JEOL, Japan).

### Rheological properties

The hydrogels were fabricated as described and the rheological properties of hydrogels in different groups were measured by a rheometer (Kinexus ultra + , Malvern, UK) at the temperature range of 25–40 °C. The heating rate was set at 5 °C /min and the angular frequency was 1 rad/s. The storage modulus (G’), loss modulus (G’’) and shear viscosity of the samples were measured accordingly. Besides, mixed EVs@FEC hydrogels were prepared by deionized water containing EVs (50 μg/ml). The rheological properties of FEC and EVs@FEC then were measured and compared.

### Evaluation of EVs release

The EVs (50 μg/ml) were added to the mixed hydrogel and soaked in PBS at 37℃. The released levels of EVs were measured by BCA protein assay (Beyotime, China). The supernatant on Days 0–7 was collected and measured the total protein levels according to the manufactural protocol. Equivalent EVs were added to the PBS and the protein level was measured and marked as initial concentration. The EVs release rate (%) was calculated by the formula: (C_1_-C_0_)/(C_0_) × 100%. C_1_ was the released protein concentration measured on different days and C_0_ was the initial concentration.

### Live/dead staining

Cell viability of NP cells in FEC hydrogels was evaluated by Calcein/PI Live/Dead Assay kit (Beyotime, China). The culture dish was coated with or without FEC hydrogels, and then the NP cells were seeded and cultured for 2 days. After removed the culture medium, the Calcein/PI solution was added and incubated for 0.5 h. NP cells were placed under a fluorescence microscope (Olympus, USA) and random field of images were captured. Dead cells (PI-positive) were quantified by ImageJ 1.52a (National Institutes of Health, USA).

### EVs labelling and uptake assay

Purified EVs were labeled with 5 μM PKH26 (Sigma-Aldrich, USA) according to the manufacturer's instructions. In order to remove unincorporated dyes, the mixture was washed in PBS and centrifuged at 110,000 × *g* for 70 min. For internalization assay, EVs (50 μg/ml) were suspended in medium and incubated with NP cells at 37 °C in the EVs group. In the EVs@FEC group, the hydrogel mixed with labelled EVs was placed in a coculture transwell to realize the sustained release. For immunofluorescence analysis, the NP cells were fixed and stained with phalloidin (Beyotime, China) for 1 h and DAPI for 5 min at specific time points. Then, the samples were placed under a fluorescence microscope (Olympus, USA) for image capture. Uptake of EVs was assessed by mean fluorescence intensity (MFI) of red fluorescent signal. For labelled EVs in hydrogels, the hydrogels were transferred to a confocal dish and images were captured via a confocal microscope (Nikon A1R SI Confocal, Japan). For internalization assay through flow cytometry, the treated cells were collected and then applied to FACSCalibur flow cytometer (BD Biosciences, USA). The positive rate of labelled cells was analyzed by FlowJo X software (Tree Star, USA).

### Ex vivo experiments

All the animal experiments were approved by the Animal Experimentation Committee of Huazhong University of Science and Technology. For ex vivo culture, caudal discs with endplates were isolated from Sprague–Dawley rats (male, 8 weeks old). The osmolarity of the culture medium was adjusted to 400 mOsm by the solution (1.5% of a 5 M NaCl and 0.4 M KCl) as previously described [14, 28]. All the discs were incubated under a hypoxic atmosphere (37 °C, 5% O_2_) with saturated humidity. The discs were cultured with TNF-α (50 ng/mL) to initiate disc degeneration slowly. Then, the discs were injected with PBS (IDD group), or EVs (2 μL, 5 μg/μL) weekly, or FEC (2 μL), or EVs@FEC (2 μL with 10 μg EVs) using 33-gauge needle (Hamilton, Benade, Switzerland). The culture medium was replaced once every three days.

### In vivo experiments

Sprague–Dawley (male, 8 weeks old) rats were purchased from the Experimental Animal Center of Tongji Medical College, Huazhong University of Science and Technology. A surgical model of IDD was conducted by needle puncture as previously described [14]. The discs of rat (Co 6/7, 7/8, and 8/9) were marked by palpation and verified by radiography. The Co 6/7 was set as sham disc which was punctured with the 33-gauge needle, and Co 7/8 was degenerated disc which was induced by a 20-gauge needle. The Co 8/9 disc were injected with EVs (2 μL, 5 μg/μL) weekly, or FEC (2 μL), or EVs@FEC (2 μL with 10 μg EVs) using 33-gauge needle.

### Radiological examination

The rats or discs were conducted with radiography and fluorescence imaging using an In-Vivo MS FX PRO imaging system (Bruker, USA). The fluorescence intensity of each disc was quantified by Bruker MI software. Besides, images under the X-ray model were also captured. The disc height was measured and the disc height index (DHI) was calculated as previously described [29]. Briefly, the change of DHI was used to evaluate disc degeneration and calculated according to the formula: DHI % = post-DHI / pre-DHI × 100%. Post-DHI was the post-operation DHI and pre-DHI was the pre-operation DHI. Magnetic resonance imaging (MRI) was performed using a MRI system (BRUKER BioSpec, Germany), and sagittal T2-weighted images were used to assess the signal of the discs, indicating the change of water content. Pfirrmann grades based on the T2-weighted section images were used to evaluate the degree of IDD as previously described [29].

### Histological analysis

The discs were collected at specific time points and then fixed in 4% formaldehyde, and decalcified slowly and steadily using EDTA (0.5 M, Servicebio, China). After dehydrated and embedded in paraffin, the paraffin blocks were cut into 4-μm slices in the coronal plane. These slices were stained with hematoxylin and eosin (HE), Safranin O-fast green (S–O), or Masson. The degenerative degree of discs was evaluated by a histological grading scale [13]. This scale was based on 5 categories of disc changes: with 0 points for a normal disc and 15 points for a severely degenerated disc.

### Statistical analysis

Data are presented as mean ± standard deviation (SD). All experiments were performed independently, at least in triplicate. Student’s t-test was used for comparisons between two groups. For multiple group comparisons, one-way or two-way analysis of variance (ANOVA) with Tukey’s post hoc test was used. Statistical significance was measured using the GraphPad Prism 8 software (La Jolla, CA, USA), with the statistical significance threshold set at P < 0.05 (*P or ^#^P < 0.05, **P or ^##^P < 0.01, ***P or ^###^P < 0.001).

## Results

### The effect of MSC-derived EVs on human NP cells

**Fig. 2:**
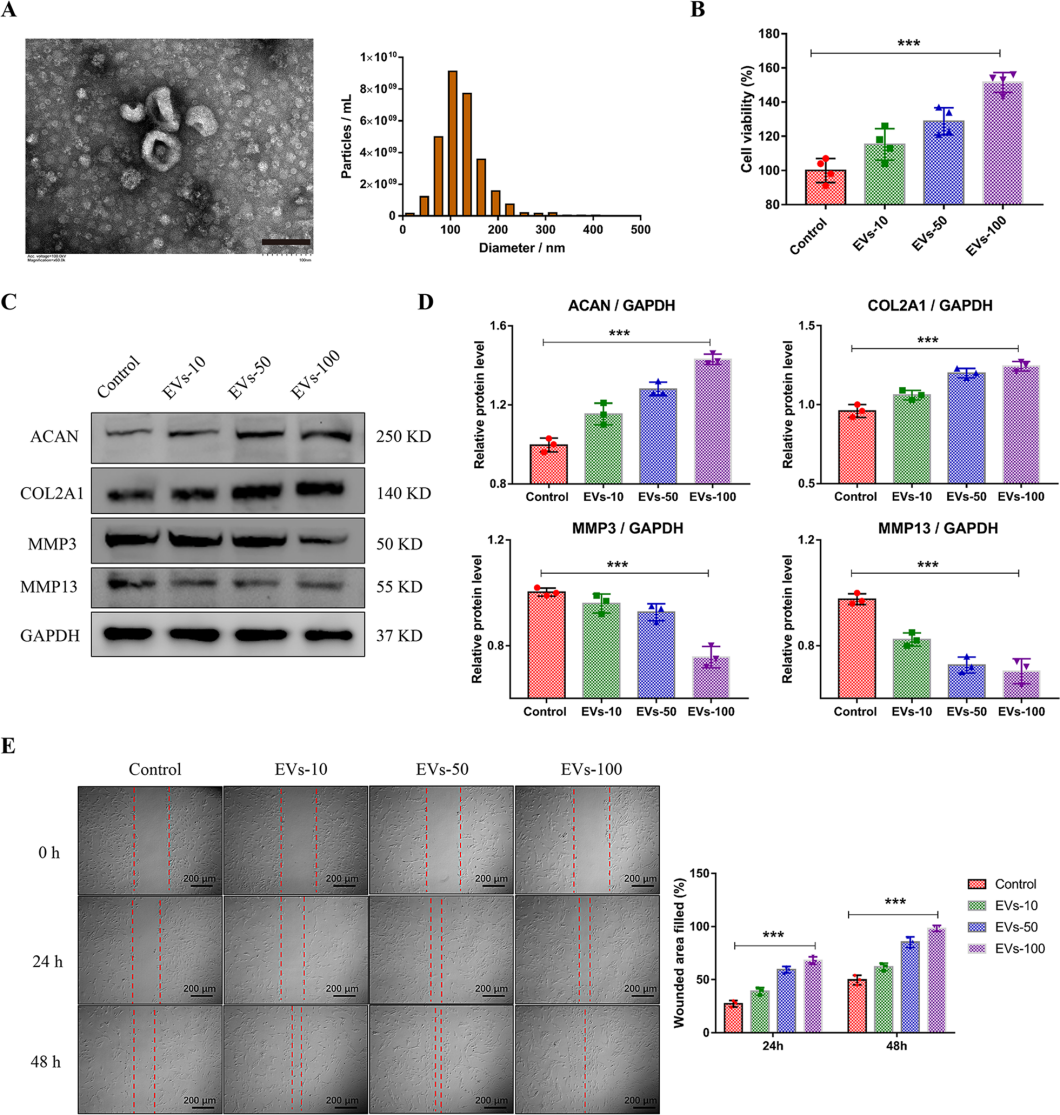
MSC-EVs promoted the anabolic metabolism, proliferation and migration of NP cells. **A** Morphology of EVs observed by transmission electron microscopy (left panel) and diameter distribution evaluated by nanoparticle tracking analysis (right panel). Scale bar: 100 nm. **B** NP cells were treated with 10 μg/ml (EVs-10), 50 μg/ml (EVs-50), or 100 μg/ml (EVs-100) EVs for 24 h. Cell viability was evaluated by CCK-8 assay. **C** Expression of ACAN, COL2A1, MMP3, and MMP13 measured by western blot and the quantification of these protein levels (**D**). **E** Representative images of the wound healing assay in NP cells. The percentages of filled wounded area were quantified (right panel). Data were presented as mean ± SD of three independent replicates. ***P < 0.001

Small EVs obtained from MSCs-cultured medium were characterized by size and morphologic analysis. The double-layer membrane of EVs was shown in the TEM images and NTA analysis also revealed that the diameter of EVs was approximately from 30 to 150 nm (Fig. 2A). Several studies have indicated a decreased number of viable resident cells and catabolic ECM metabolism during IDD [23]. We then investigated whether MSC-EVs affect these phenotypes of NP cells. After incubated with NP cells, these EVs increased the cell viability of NP cells in a dose-dependent manner (Fig. 2B). To evaluate the effect of EVs on ECM metabolism, the levels of aggrecan (ACAN) and type II collagen (COL2A1), and matrix degrading enzymes MMP3 and MMP13, were measured accordingly (Fig. 2C). Treatment of EVs significantly promoted the expression of ACAN and COL2A1, and decreased the catabolic molecules both in mRNA levels (Additional file 1: Fig. S1) and protein levels (Fig. 2D). Besides, wound healing assay indicated that EVs could promote the migration of NP cells in vitro (Fig. 2E). These results showed that MSC-EVs promote the proliferation, migration and anabolic metabolism profile of NP cells.

### EVs deliver vasorin to mediate the effect on NP cells

**Fig. 3:**
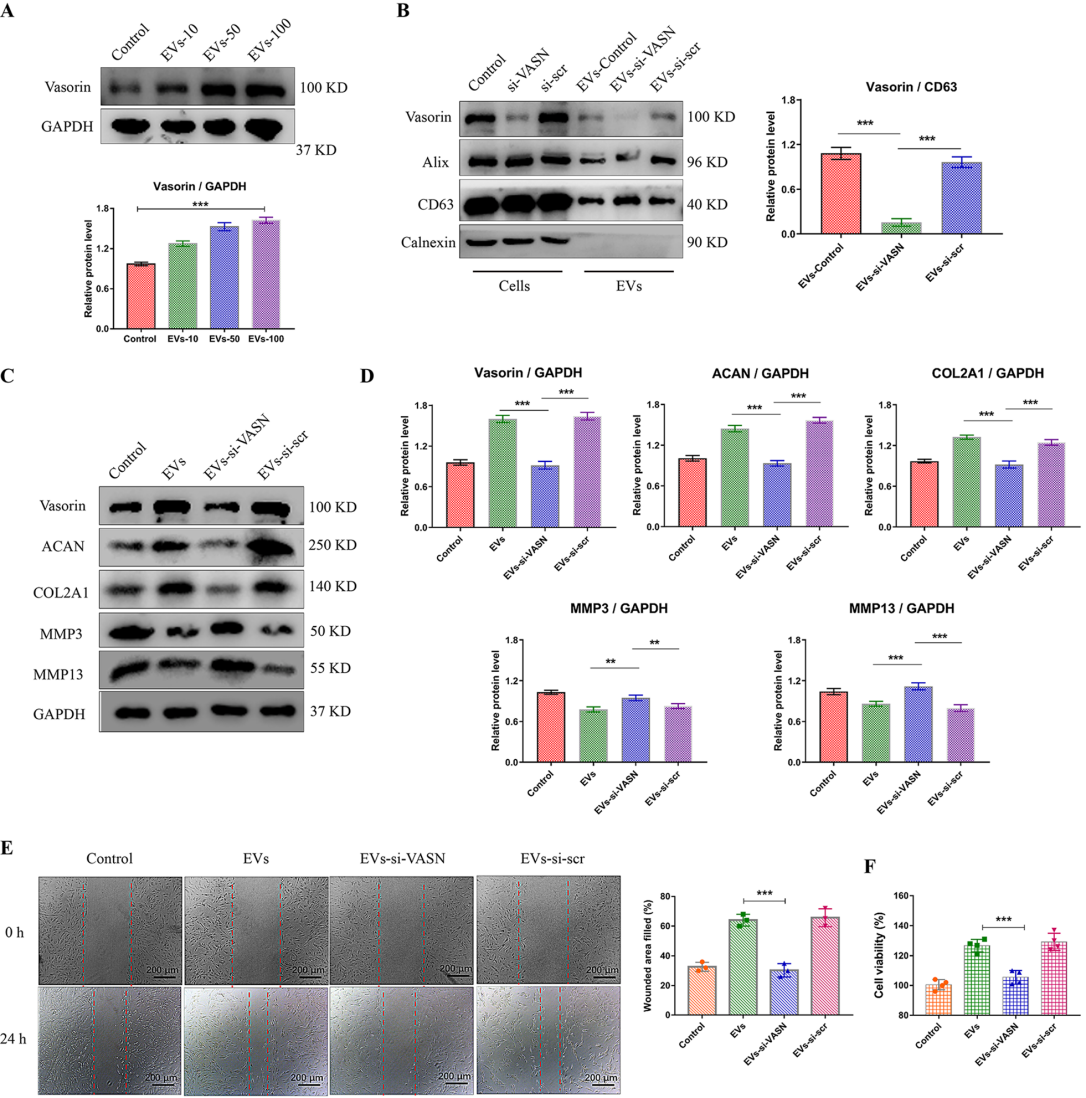
Vasorin in EVs promoted the anabolic metabolism, proliferation and migration of NP cells. **A** NP cells were treated with 10, 50, or 100 μg/ml EVs for 24 h. The protein level of Vasorin was measured and quantified by western blot. **B** EVs were isolated from common MSCs (EVs-control), siRNA-VASN transfected MSCs (EVs-si-VASN) or scrambled siRNA transfected MSCs (EVs-si-scr). The protein levels of Vasorin, Alix and CD63 were measured in the MSCs and EVs fraction. The relative levels of vasorin in EVs (relative to CD63) was quantified (right panel). **C** NP cells were incubated with these EVs equivalently (50 μg/ml) for 24 h. The protein levels of Vasorin, ACAN, COL2A1, MMP3 and MMP13 were measured and quantified in NP cells (**D**). **E** Representative images showed the wound healing assay of NP cells treated with different kinds of EVs for 24 h. The percentages of filled wounded area were quantified (right panel). **F** The NP cell viability in different group was evaluated by CCK-8 assay. Data were presented as mean ± SD of three independent replicates. P > 0.05 (*ns* not significant), **P < 0.01, and ***P < 0.001

We intended to investigate the specific mechanism of EVs-mediated effects on NP cells. We then wondered whether Vasorin expressed in MSC-EVs mediates the effects on NP cells. Firstly, we measured the protein levels of Vasorin in MSCs, MSC-EVs and the left culture medium. It showed that Vasorin was rich in EVs fraction (Additional file 1: Fig. S2A). After NP cells were incubated with EVs, the levels of Vasorin in NP cells increased with the concentration of added EVs (Fig. 3A). Then, we utilized siRNA for VASN to knockdown the expression of Vasorin in MSCs (Additional file 1: Fig. S2B). We therefore obtained the EVs with significantly decreased expression of Vasorin (EVs-si-VASN) (Fig. 3B). When NP cells were incubated with This kind of EVs, the therapeutic effect mediated by EVs was mostly abrogated. Compared with the control EVs, the EVs-si-VASN could not elicit significantly elevated expression of ACAN and COL2A1 in NP cells (Fig. 3C, D). The results in wound healing assay and cell viability assay also supported that EVs with little Vasorin expression could not promote the proliferation and migration of NP cells (Fig. 3E, F). These data revealed that Vasorin probably mediated the therapeutic effect of EVs on NP cells.

### EVs activate the Notch1 signaling pathway through transferring Vasorin

**Fig. 4:**
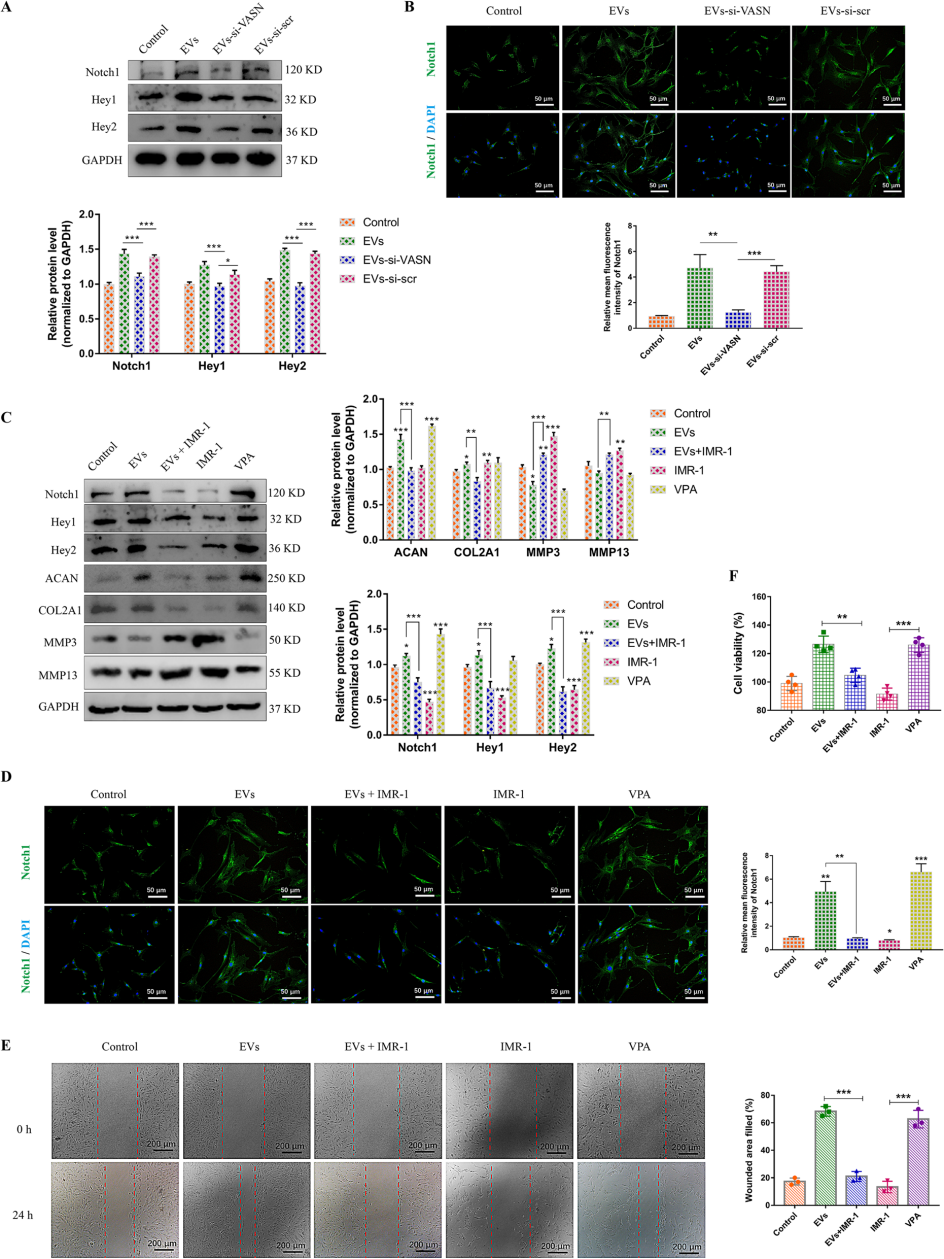
Pharmacological inhibition of Notch1 signaling impeded the EVs-mediated effects on NP cells. **A** NP cells were treated with 50 μg/ml common EVs, EVs-si-VASN (isolated from si-VASN transfected MSCs) or EVs-si-scr (isolated from si-scr transfected MSCs) for 24 h. The expression levels of Notch1, Hey1 and Hey2 were measured and quantified. **B** Representative images of Notch1 fluorescence in NP cells and the quantification of mean fluorescence intensity. **C** NP cells were treated with EVs (50 μg/ml), IMR-1 (10 μm), valproic acid (VPA, 100 μm), or EVs combined with IMR-1. The expression levels of Notch1, Hey1, Hey2, ACAN, COL2A1, MMP3 and MMP13 were measured and quantified. **D** Representative images of Notch1 fluorescence in NP cells and the quantification of mean fluorescence intensity. **E** Representative images of Wound healing assay and the percentages of filled wounded area were quantified. **F** The NP cell viability in different group was evaluated by CCK-8 assay. Data were presented as mean ± SD of three independent replicates. P > 0.05 (ns, not significant), *P < 0.05, **P < 0.01, and ***P < 0.001

Although initially discovered as a TGF-β signaling regulator [30], Vasorin has been indicated to bind Notch1 and regulate its turnover [31]. Notch1 signaling is associated with intervertebral disc hypoxia environment and closely related to cell proliferation and senescence [32, 33]. We then investigate the role of Notch1 in Vasorin-mediated effect on NP cells. After NP cells were incubated with equivalent EVs and EVs-si-VASN, the expression levels of Notch1 and the Notch downstream targets, including Hey1 and Hey2 were measured (Fig. 4A). These results, also including immunofluorescence analysis of Notch, indicated that the low expression level of Vasorin in EVs could not activate Notch1 signaling in NP cells (Fig. 4B). Furthermore, we treated NP cells with the Notch1 signaling activator, valproic acid (VPA) and Notch1 signaling inhibitor, IMR-1. Like EVs treatment, VPA could induce the expression of Notch1, Hey1, and Hey2. However, IMR-1 significantly inhibit Notch1 signaling, ACAN and COL2A1 levels, and this effect could not be rescued by EVs incubation (Fig. 4C). The effects of EVs on NP cell proliferation and migration were also abrogated by the inhibition of Notch1 signaling (Fig. 4D, E).

**Fig. 5:**
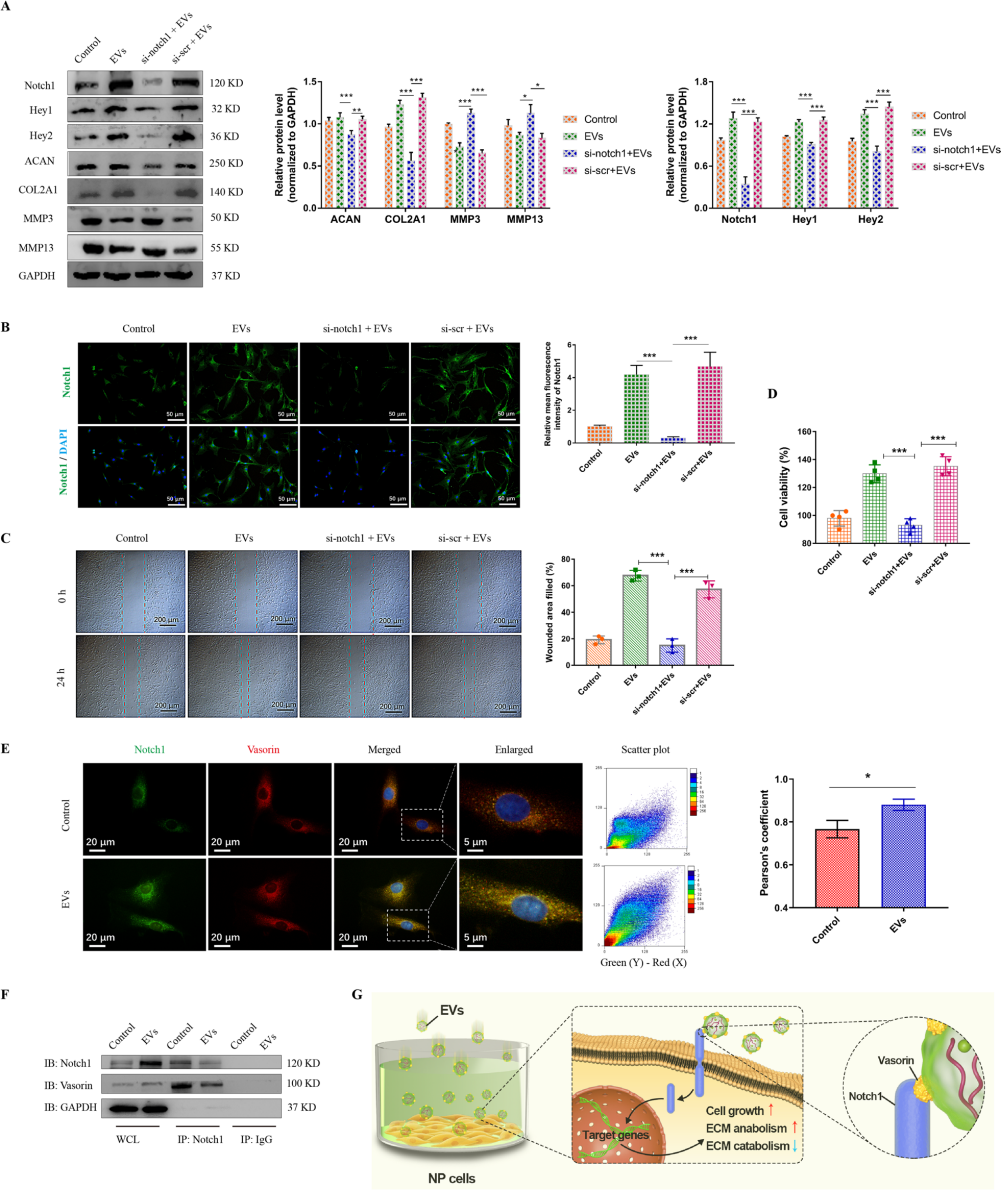
Knockdown of Notch1 signaling impeded the EVs-mediated effects on NP cells. **A** NP cells were transfected with siRNA targeted for Notch1 (si-Notch1) or a scrambled siRNA (si-scr), and then treated with EVs (50 μg/ml) for 24 h. The expression levels of Notch1, Hey1, Hey2, ACAN, COL2A1, MMP3 and MMP13 were measured and quantified. **B** Representative images of Notch1 fluorescence in NP cells and the fluorescence intensity. **C** Representative images of Wound healing assay and the percentages of filled wounded area were quantified. **D** The cell viability in different group was evaluated by CCK-8 assay. **E** Representative immunofluorescent images of Vasorin (green) and Notch1 (red) in NP cells. Nuclei were stained with DAPI (blue). The colocalization analysis of Pearson coefficient was quantified (right panel). **F** Immunoprecipitation (IP) of Notch1 in NP cells treated with or without EVs and blotted for Notch1 and Vasorin. IgG was used as a negative control. **G** Mechanism schemes showed the EVs-mediated effects on NP cells. Data were presented as mean ± SD of three independent replicates. P > 0.05 (ns, not significant), *P < 0.05, **P < 0.01, and ***P < 0.001

To further assure the role of Notch1, we knockdowned the Notch1 expression in NP cells, and found that EVs-mediated notch activation and the downstream effects were inhibited by siRNA (Fig. 5A). The immunofluorescence analysis also indicated the change of Notch signaling (Fig. 5B). Similarly, the effects of EVs on NP cell proliferation and migration were also abrogated by the Notch1 knockdown (Fig. 5C, D). Therefore, it was most likely that Vasorin in EVs affects NP cells by activating the Notch1 signaling pathway. We then evaluated the integration between Vasorin and Notch1. The results based on and immunofluorescence analysis showed that the colocalization of Vasorin and Notch1 in NP cells (Fig. 5E). The immunoprecipitation also revealed the integration of vasorin and Notch1 (Fig. 5F). These results demonstrated that Vasorin delivered by EVs activated Notch1 signaling and mediated the therapeutic effects on NP cells (Fig. 5G).

### Characterization of FEC hydrogel and EVs encapsulation

**Fig. 6:**
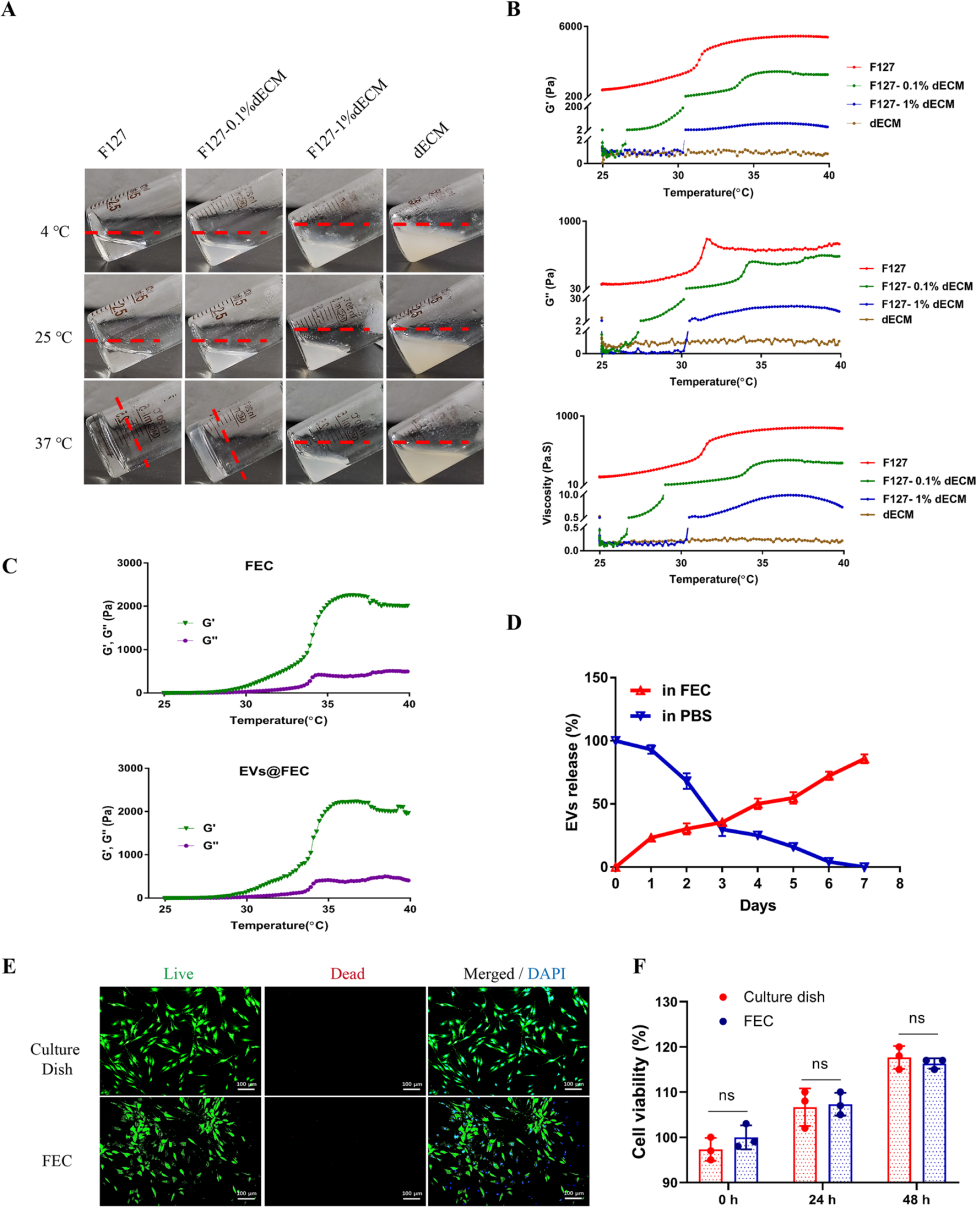
Fabrication and characterization of FEC hydrogel. **A** Images of F127, F127 with 0.1% or 1% dECM and dECM in tilted bottle at 4, 25, 37 °C. **B** Variation of storage modulus G’, loss modulus G’’ and viscosity of hydrogels as the temperature changes. **C** EVs (50 μg/ml) were encapsulated into FEC hydrogel (EVs@FEC), and the G’ and G’’ were evaluated by a rheometer. **D** The protein level of released EVs in FEV hydrogel was measured by BCA protein assay, and compared with the degradation rate in PBS. **E** Live/Dead staining of NP cells cultured on the FEC hydrogel and common culture dish. **F** The cell viability of NP cells cultured in common dish or FEC was evaluated by CCK-8 assay. Data were presented as mean ± SD of three independent replicates. P > 0.05 (ns, not significant)

Pluronic F127 is a well-known thermoresponsive copolymer, which can form a semi-solid gel at 37 °C [34]. Previous studies have designed many drug delivery systems based on Pluronic F127 [35–37]. In this study, we developed a composite hydrogel (FEC) as the EVs carrier which is composed of thermoresponsive Pluronic F127 and biocompatible dECM from NP tissues (Additional file 1: Fig. S3A). Decellularized ECM obtained from human NP tissues was evaluated by histological staining, that was without residual host cell nucleus (Additional file 1: Fig. S3B). Hydrogels with thermoresponsive ability is convenient for injection in IDD therapy. Therefore, we compared the different concentration FEC hydrogels and found that FEC hydrogel with 0.1% dECM showed a well sol-to-gel transition at 37℃, which is an ideal choice for our following experiments (Fig. 6A). After a freeze-drying, the FEC hydrogel showed a porous structure which allows for EVs loading (Additional file 1: Fig. S3C). To assess the mechanical properties, the rheological assay was performed and results revealed that the storage modulus (G’), loss modulus (G’’) and viscosity of 0.1% FEC was similar with F127 and much better than 1% FEC (Fig. 6B). Additionally, EVs were encapsulated into 0.1% FEC hydrogel (EVs@FEC) as described in our methods. The addition of EVs had little influence on the rheological property of FEC hydrogel (Fig. 6C). We then evaluated the EVs release efficiency of FEC hydrogel. Our results showed that EVs in PBS underwent increasing degradation, and EVs in FEC released slowly in a week in vitro (Fig. 6D). The NP cells were seeded in FEC hydrogel, and no obvious dead cells were detected (Fig. 6E). The cell viability of NP cells cultured in common dish and FEC hydrogel was without significant difference, which demonstrated the cell compatibility of FEC (Fig. 6F). These results indicated the multifunctional properties of FEC hydrogel, including thermoresponsive ability, injectability, biocompatibility, and sustained release of EVs.

### Sustained release and cellular uptake of EVs from FEC hydrogel

**Fig. 7:**
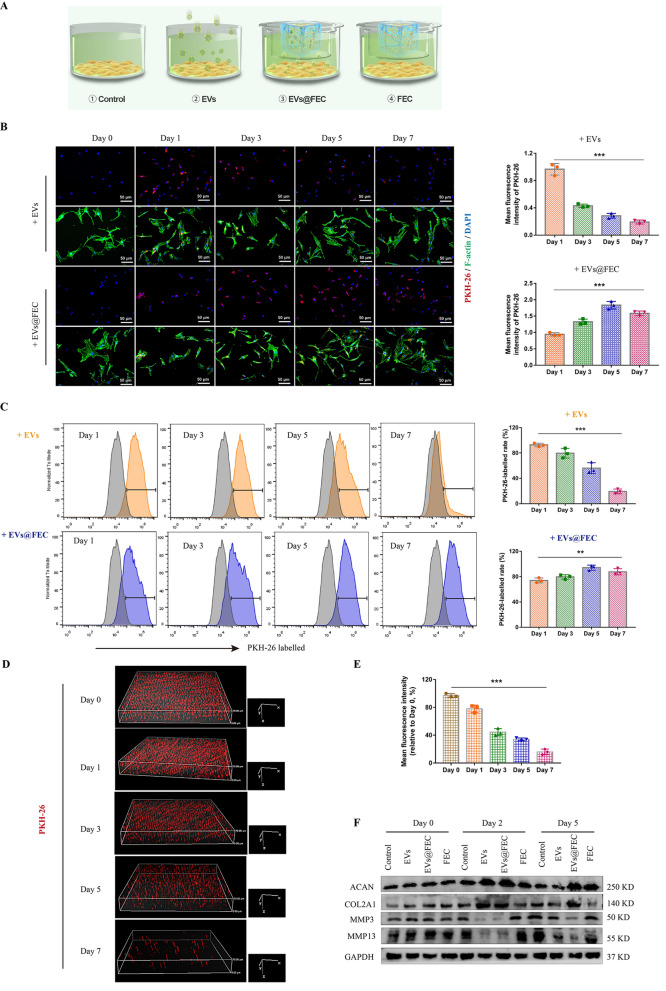
Evaluation of EVs release in FEC hydrogel and cellular uptake in NP cells. **A** Schematics showing NP cells culture with EVs, EVs@FEC hydrogel and FEC hydrogel. The pore size of transwell is 0.4 μm, allowing EVs to get through. **B** Equivalent PKH-26-labelled EVs in PBS or FEC hydrogel were cultured with NP cells for a week. Representative immunofluorescence images showed the uptake of EVs in NP cells, and the uptake rate was evaluated based on the mean fluorescence intensity (right panel). **C** Flow cytometry of NP cells indicated the uptake rate of PKH-26-labelled cells. **D** Representative confocal 3D images of PKH-26-labelled EVs in FEC hydrogel at 37℃ and the EVs retention rate was evaluated based on the mean fluorescence intensity (**E**). **F** Expression of ACAN, COL2A1, MMP3, and MMP13 measured by western blot in NP cells cultured with EVs, EVs@FEC or FEC at specific time points. Data were presented as mean ± SD of three independent replicates. P > 0.05 (ns, not significant), **P < 0.01, and ***P < 0.001

To further investigate the EVs release efficiency of FEC hydrogel, we assessed the EVs uptake in NP cells in vitro. Equivalent EVs were labelled with PKH-26, and encapsulated into FEC hydrogel or added into NP cell culture medium directly (Fig. 7A). The NP cells could internalize EVs when the EVs slowly released from FEC hydrogel, while the uptake amount deceased increasingly in the group with one-off EVs addition (Fig. 7B). The flow cytometry also showed the rate of PKH-26-labelled cells increased slowly in the EVs@FEC group (Fig. 7C). Under a confocal 3D view, we observed the PKH-26-labelled EVs decreased over time in FEC hydrogel (Fig. 7D, E). What’s more, we then evaluated the effect of EVs uptake on NP cell phenotypes. The addition of EVs into NP cells could increase the expression of ACAN, COL2A1 and decrease the MMP3 and MMP13, while this could not achieve a lasting effect. EVs in FEC hydrogel released slowly, which could keep NP cell an anabolic metabolism in a relatively long time (Fig. 7F, Additional file 1: Fig. S4A). The NP cells in the EVs@FEC group also maintained a much better viability than in the EVs group (Additional file 1: Fig. S4B). These results demonstrated that FEC hydrogel could realize a sustained EVs release and have a therapeutic potential on NP cells.

### Intervertebral disc regeneration by FEC hydrogel ex vivo

**Fig. 8:**
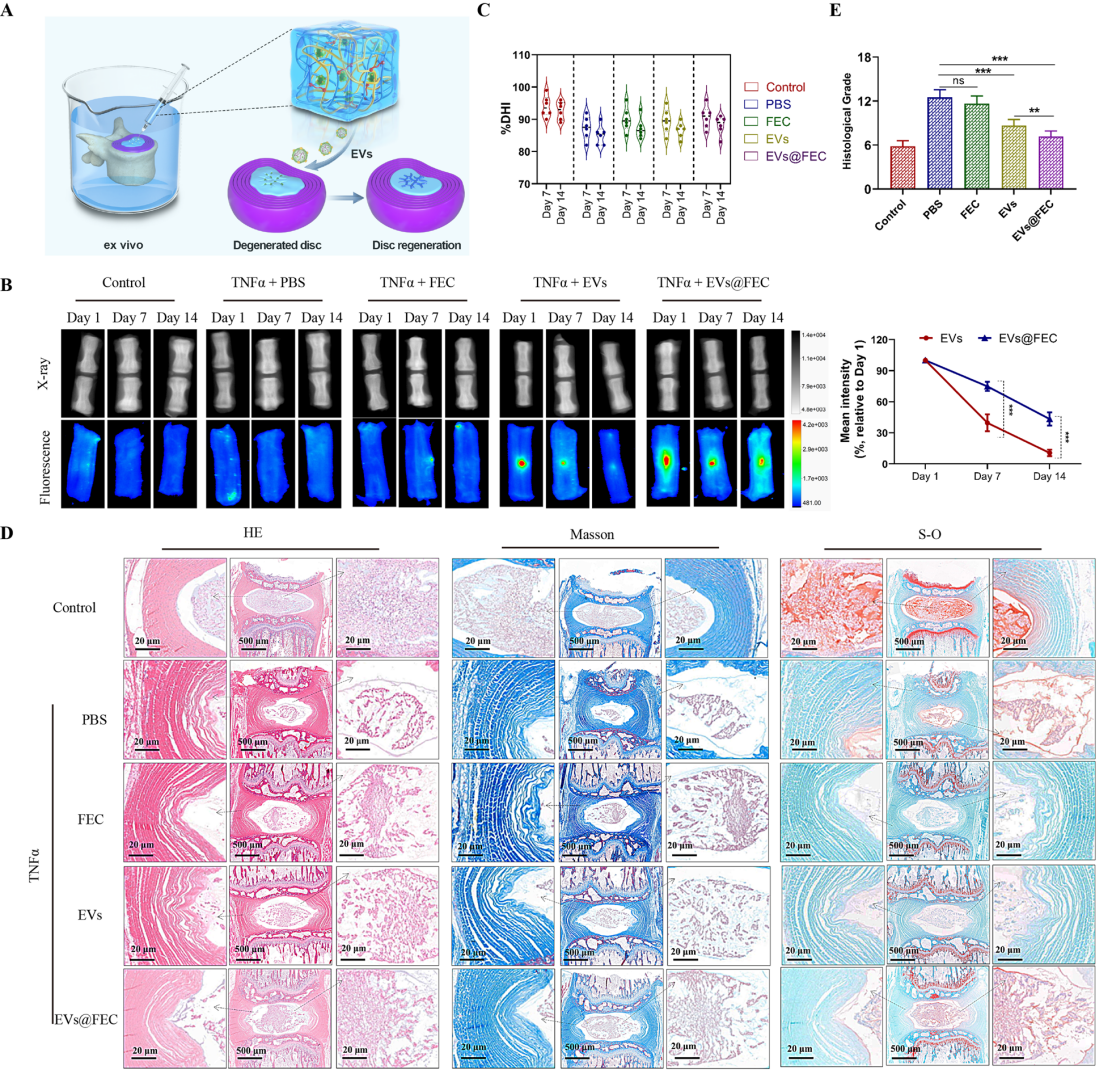
Effect of EVs in FEC hydrogel on the disc degeneration ex vivo. **A** Diagrammatic sketch of the ex vivo disc degeneration model. **B** Ex vivo imaging on X-ray and fluorescence pattern of disc at Day 1, Day 7 and Day 14 (left panel). The mean fluorescence intensity in the EVs and EVS@FEC group was quantified (right panel). **C** The %DHI of disc was calculated correspondingly. **D** Hematoxylin and eosin (HE), Safranin O-fast green (S–O), and Masson staining of disc organ in each group. **E** Histological grades of disc based on histological staining. Data were presented as mean ± SD of six independent replicates. P > 0.05 (ns, not significant), *P < 0.05, **P < 0.01, and ***P < 0.001

We constructed an ex vivo rat disc degeneration model as previously described [14]. The isolated disc organ was cultured in medium, and the inflammatory cytokine TNF-α was used to induce disc degeneration (Fig. 8A). The fluorescence images of discs were captured to reveal the EVs retention in disc. We observed that EVs in hydrogel degraded more slowly than the EVs with one-off injection (Fig. 8B). The change of DHI index reveals the change of disc space indicating the degree of disc degeneration. We found that the change of DHI index was without significance between PBS and EVs group in the ex vivo model (Fig. 8C). It was indicated that the disc space height has no indication for disc degeneration in the ex vivo model.

Besides, the histological evaluation of these discs showed the morphology, cellularity, fiber structure and proteoglycan distribution of disc tissues (Fig. 8D). The degenerative degree of discs was then assessed based on a histological grading scale. The results indicated that both EVs and EVs@FEC decreased the degenerative score of disc degeneration, while the FEC alone made no affect on disc degeneration (Fig. 8E). The histological score of the EVs@FEC group was also lower than in the EVs group, indicating a better therapeutic effect of EVs@FEC. These results revealed that EVs@FEC could effectively ameliorate the disc degeneration ex vivo and this regenerative effect seems more promising than the direct EVs injection.

### Intervertebral disc regeneration by FEC hydrogel in vivo

**Fig. 9:**
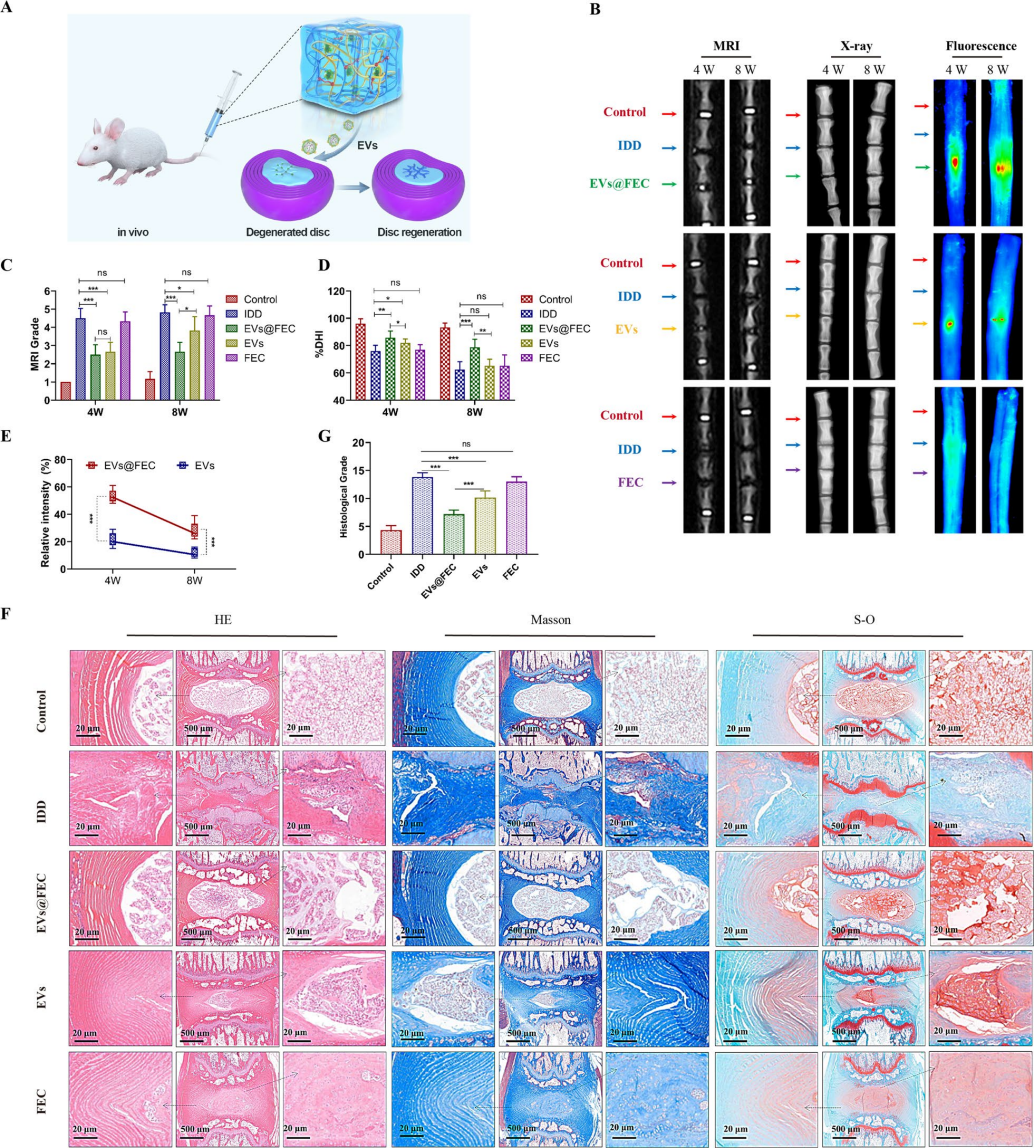
Application of EVs in FEC hydrogel in disc degeneration in vivo. **A** Diagrammatic sketch of the in vivo disc degeneration model. (B) The MRI, X-ray and fluorescent images of rat tail in different groups at weeks 4 (4 W) and weeks 8 (8W). **C** The MRI grade was assessed based on the MRI images. **D** The %DHI of disc was calculated based on the X-ray images. **E** The mean fluorescence intensity in the EVs and EVS@FEC group was quantified based on the fluorescent images. **F**, **G** Hematoxylin and eosin (HE), Safranin O-fast green (S–O), and Masson staining of the disc in different groups **F** and the corresponding histological grades (**G**). Data were presented as mean ± SD of six independent replicates. P > 0.05 (ns, not significant), *P < 0.05, **P < 0.01, and ***P < 0.001

To further investigate the effect of EVs in FEC hydrogel, we then utilized the in vivo rat disc degeneration model. The larger needle puncture was used to initiate disc degeneration and then followed by the EVs therapy (Fig. 9A). The discs were conducted with radiological and histological examination at 4 weeks and 8 weeks post-operative. The T2-weighted MRI images indicated the water content of disc, and the X-ray images revealed the disc height and vertebral structure (Fig. 9B). Degenerated disc was characterized by loss of water content and collapse of disc space. Our results showed that MRI grades in the EVs@FEC and EVs group were much lower and better than in the IDD group (Fig. 9C). The change of DHI was also much less in the EVs@FEC and EVs group than in the IDD group (Fig. 9D). We also observed that the EVs@FEC group was with a better MRI and X-ray result than in the EVs group, indicating a better therapeutic effect of EVs@FEC.

Besides, the fluorescence analysis showed the retention of EVs in the disc (Fig. 9B). According to the mean fluorescent intensity, it was also revealed that the vesicle retention rate was higher in the EVs@FEC group than in the EVs group (Fig. 9E), although the difference between the two groups gradually decreased as time went. Besides, histological staining was used to evaluated the degree of disc degeneration (Fig. 9F). The analysis based on the histological staining found that both the EVs@FEC and EVs treatment decreased the histological grade compared with the IDD group. We also observed that EVs@FEC treatment achieved a better histological grade of disc than in the EVs group. In all, these results demonstrated that EVs delivered by FEC hydrogel could retard the progression of IDD more efficiently in vivo.

## Discussion

Accumulating evidence has revealed the regenerative effect of MSC-derived EVs on degenerative musculoskeletal diseases [38]. However, the rapid clearing and degradation of EVs hinder their applications during the local or systemic delivery therapy. Bioactive hydrogels could increase the tissue retention of EVs and serve as an EVs controlled release platform, which has promising potentials in tissue regeneration [39]. In the present study, we developed a thermoresponsive hydrogel composed of F127 and decellularized NP tissues with fine biocompatibility, which allows for convenient EVs injection and sustained EVs releases during IDD therapy. In addition, our results illuminated the therapeutic mechanism of EVs on NP cells, that EVs-derived Vasorin induced the activation of Notch signaling pathway, thereby promoting cell proliferation and a healthy ECM metabolic pattern. In short, our study focuses on EVs delivery vehicles and aims to improve EVs therapeutic performance in IDD therapy.

Stem cells have shown therapeutic promise for IDD in basic and preclinical studies [6]. Diminishing of viable resident cells is one important feature of IDD. Delivery of stem cells could supplement NP-like cells through their differentiation [40]. Besides, the increasingly loss of matrix proteins around the NP area, such as proteoglycans, is also an evident structural symbol of IDD, indicating a catabolic ECM metabolism. Exogenous stem cells could modulate inflammation mainly through the paracrine effect to keep a healthy ECM metabolism profile during IDD [40]. Recent studies also investigate the existence of endogenous disc-derived progenitor cells and their application in IDD therapy [41]. However, the main obstacle of stem cell therapy in IDD is whether the cells could adapt the progressively degenerated disc microenvironment, including mechanical stress, osmotic stress, acidic and hypoxia features. Importantly, EVs could serve as an alternative to stem cells in IDD therapy, which may solve the survival problem of the transplanted cells [42]. Delivery enough amount of EVs via suitable carriers could also exert the effect of cell therapy.

EVs are important components mediating the paracrine effect of stem cells. Compared to cell therapy, EVs fraction are convenient in preservation and transport, which could be utilized promptly upon thawing [11]. Moreover, EVs isolated from culture medium could achieve sufficient and stable production under the in vitro culture conditions [11]. Collectively, EVs-based tissue regeneration may realize a more simplified procedure and lower manufacturing cost compared to cell therapy. Up to now, delivery of EVs for therapy is mainly via systemically intravenous injection or direct local injection [43]. How to increase the retention of EVs in injury sites and decrease the degradation of EVs has become the important issue in EVs-based therapy. In the present study, we used a thermoresponsive hydrogel as the EVs carrier, providing a promising strategy for EVs-based IDD therapy. Utilization of hydrogel permits the sustained release of EVs and decreases the degradation rate, which is in favor of a better therapeutic potency [44–46]. Hydrogel composed of natural biomaterials, such as collagens and hyaluronan, could provide biomimetic environments for bearing cells or target cells [47]. Decellularized ECM contains various kinds of natural matrix that serves as an ideal deliver carrier for EVs. We used the synthetic copolymer F127 and the NP tissue-derived decellularized ECM to obtain a composite hydrogel for EVs delivery. This composite hydrogel presented a good biocompatibility and allowed for rapid gelation after local injection. Our previous studies and current study have indicated the positive role of EVs in IDD. However, the degradation of EVs always occurs during the application of EVs. Based on the hydrogel-based delivery route we developed, it realizes the EVs sustained release and augments their potency in IDD therapy.

Growing evidence has investigated the therapeutic efficacy of EVs in IDD [48–50]. Xing et.al. found that adipose stem cell-derived EVs regulates the expression of matrix metalloproteinases in NP cells and decreases the cell pyroptosis [46]. EVs were also indicated to inhibit the activation of inflammasome to exert anti-oxidant and anti-inflammatory effects [48]. According to these studies, it is reasonable to assume that EVs plays multifunctional roles in IDD therapy and diverse mechanism may be involved in this process. EVs may delivery various proteins, mRNAs or miRNAs to recipient cells and alter the functional status of these cells [51]. We identified a function protein of EVs, Vasorin, and indicated that EVs regulate the ECM metabolism and promote the NP cell growth and migration via Vasorin-Notch1 signaling pathway. Due to the avascular trait and poor self-healing capacity of IVD organ, the important point during IDD regeneration is to rescue the injury resident cells and promote the endogenous repair processes [52]. Delivery of EVs helps the NP cells to reconstruct a healthy ECM metabolism profile, and activates the cell growth and migration, which may be beneficial for the activation of disc endogenous repair. However, the molecular mechanisms of EVs involved in IDD regeneration are complicated and still need further investigations.

Notch signaling, an evolutionarily conserved pathway, is closely related to cell senescence and rejuvenation [53]. In a developmental perspective, notch signaling plays a role in the segmentation of the notochord sheath and the formation of spine [54]. Several reports have indicated the effect of notch signaling during the progression of IDD [55–57]. Zheng et.al. found that induction of notch signaling promoted the NP cell proliferation and induced the expression of matrix anabolic genes [57], which is consistent with our results. In the hypoxia environment of disc, the notch signaling activates and increases the target genes that maintains cell proliferation and inhibits cell differentiation [55]. Our study also verified the interaction between Notch1 and Vasorin, which is consistent with the Man’s research [31]. We found that Vasorin in EVs binds to Notch1 and activates the notch signaling pathway. Additionally, EVs may be engulfed into NP cells and released by endosomes, then directly induced the notch signaling [58]. The Vasorin-Notch1 interaction may serve as a new mechanism of EVs-related notch signaling activation.

There still have some defects in the present study. Firstly, although the F127 composite hydrogel display a good biocompatibility, the mechanical properties and water content of the synthetic hydrogel could not imitate the natural disc tissues perfectly. The addition of decellularized ECM also increase the possibility of an immune response, though it was not observed in our present study. Secondly, the Vasorin-Notch1 signaling did play a role in the effect of EVs-mediated therapy. While the pathology of IDD is complicated, the diverse mechanisms involved in IDD and disc regeneration still need further studies to investigate. Finally, although the ex vivo model and in vivo model we used in the present study are well-constructed models for disc degeneration research, we may provide the more convincing evidence if the effects were testified in the compression-induced or metabolites-induced disc degeneration model [59, 60]. Besides, the evidence will be more convincing based on the upright walking animals, such as pig and goat, which are similar to human intervertebral discs [61].

## Conclusion

In summary, we found that vasorin-containing EVs promoted the proliferation, migration and anabolism of NP cells via the Notch1 signaling. The biocompatible and thermoresponsive FEC hydrogel was fabricated and utilized to sustainably deliver EVs for intervertebral disc regeneration. The results showed that the EVs combined with FEC hydrogel presented a more satisfactory effect than one-off delivery. Therefore, EVs with FEC hydrogel have great potential in intervertebral disc regeneration and may serve as the therapeutic approach in disc injury and degeneration.

## Supplementary Information

**Additional file 1. Figure S1.** MSC-EVs promoted the anabolic metabolism of NP cells. **Figure S2.** The expression of Vasorin in EVs. **Figure S3.** Fabrication and characterization of FEC hydrogel.** Figure S4.** NP cells were cultured with EVs, EVs@FEC or FEC at specific time points. **Table S1.** Primers of targeted genes. **Table S2.** Sequences of siRNAs.

## Data Availability

All data generated during this study are included in this article.
