# Supplementary material for: Vasorin-containing small extracellular vesicles retard intervertebral disc degeneration utilizing an injectable thermoresponsive delivery system
Source: J Nanobiotechnology. 2022 Sep 19;20:420. doi: 10.1186/s12951-022-01624-1 (PMC9484164; doi:10.1186/s12951-022-01624-1)
Supplement: Supplementary file 1 — Additional file 1. Figure S1. MSC-EVs promoted the anabolic metabolism of NP cells. Figure S2. The expression of Vasorin in EVs. Figure S3. Fabrication and characterization of FEC hydrogel. Figure S4. NP cells were cultured with EVs, EVs@FEC or FEC at specific time points. Table S1. Primers of targeted genes. Table S2. Sequences of siRNAs. [file 12951_2022_1624_MOESM1_ESM.docx]

**Additional file 1**

**Vasorin-containing small extracellular vesicles retard intervertebral disc degeneration utilizing an injectable thermoresponsive delivery system**

Zhiwei Liao**^#^**, Wencan Ke**^#^**, Hui Liu**^#^**, Bide Tong**^#^**, Kun Wang, Xiaobo Feng, Wenbin Hua, Bingjin Wang, Yu Song, Rongjin Luo, Huaizhen Liang, Weifeng Zhang, Kangcheng Zhao*****, Shuai Li*****, Cao Yang*****

Department of Orthopaedics, Union Hospital, Tongji Medical College, Huazhong University of Science and Technology, Wuhan 430022, China

**#** These authors contributed equally to this work

**Additional file 1 figures：**


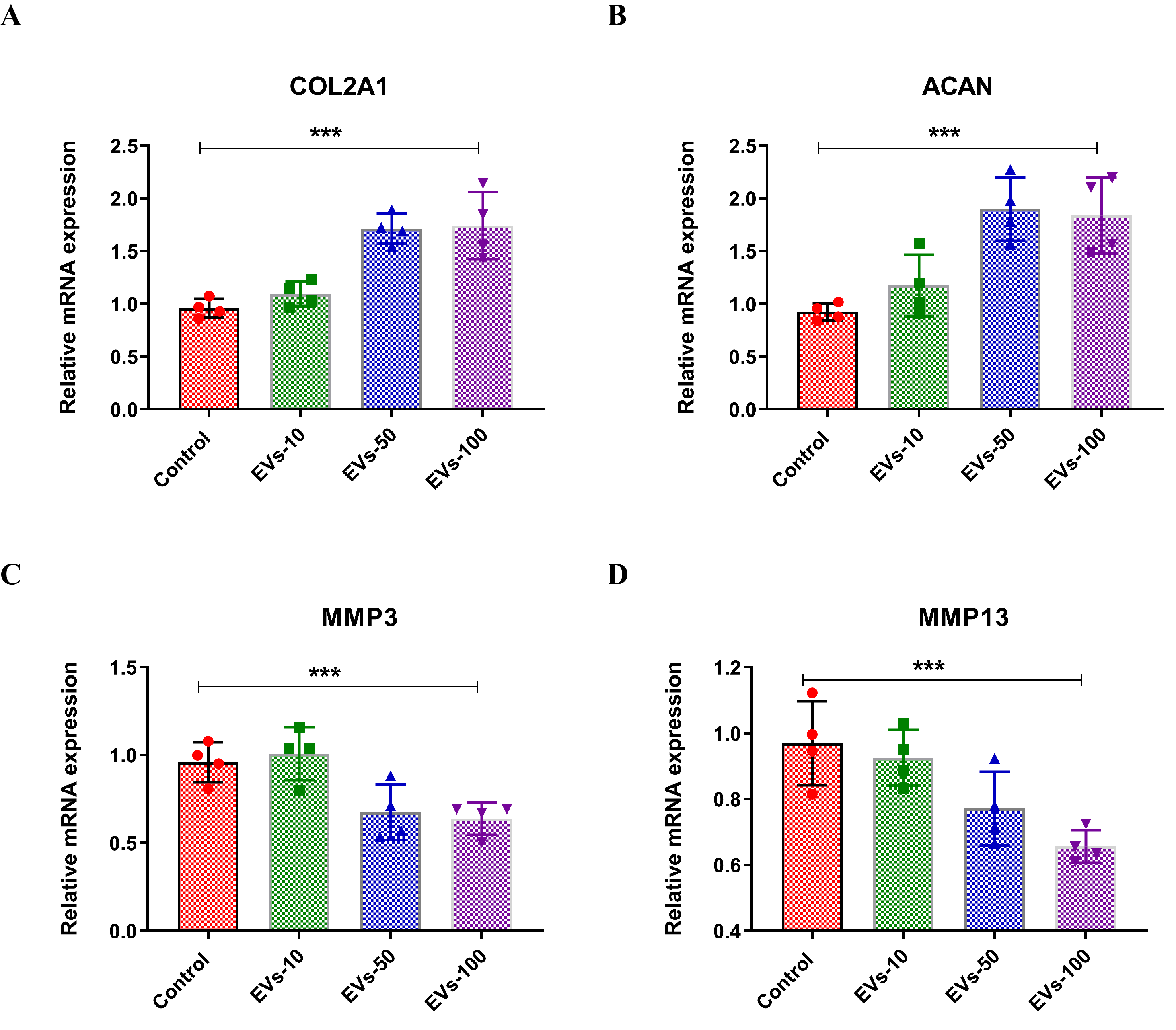


**Figure S1:** MSC-EVs promoted the anabolic metabolism of NP cells. NP cells were treated with 10 μg/ml (EVs-10), 50 μg/ml (EVs-50), or 100 μg/ml (EVs-100) EVs for 24h. The mRNA levels of COL2A1, ACAN, MMP3, and MMP13 measured by qRT-PCR (A-D). Data were presented as mean ± SD of three independent replicates. ***P < 0.001.


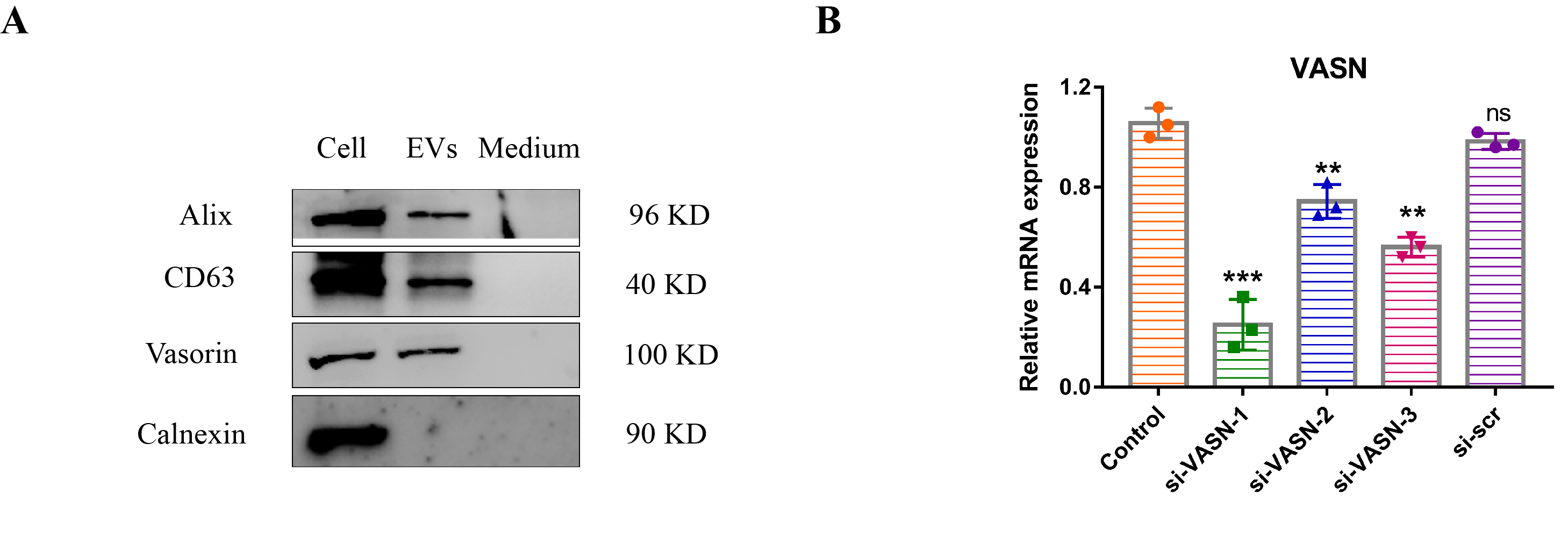


**Figure S2:** The expression of Vasorin in EVs. (A) Vasorin expression in MSC cell lysates, MSC-EVs fraction and EVs-depleted culture medium. (B) Knockdown efficiency of siRNAs targeted for VASN mRNA level. Data were presented as mean ± SD of three independent replicates. P > 0.05 (ns, not significant), **P < 0.01, and ***P < 0.001.


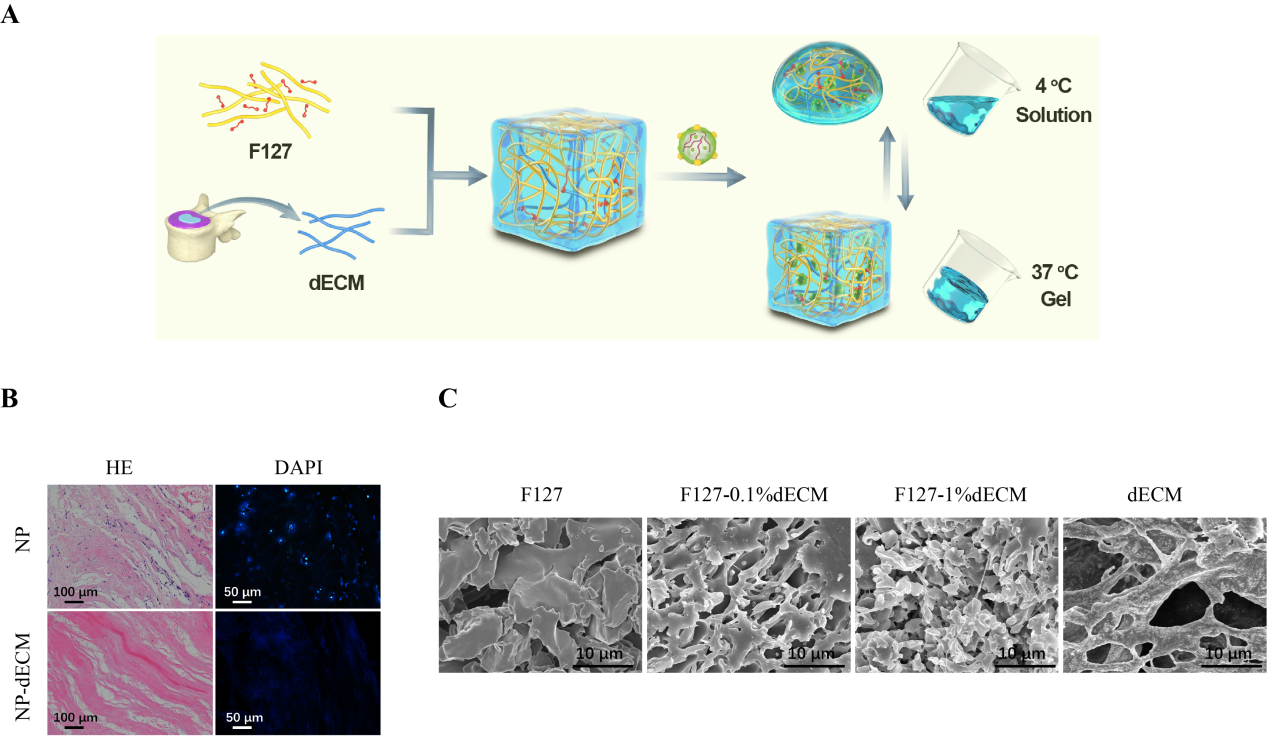


**Figure S3:** Fabrication and characterization of FEC hydrogel. (A) Workflow of hydrogel fabrication. (B) Hematoxylin-eosin (HE) and DAPI staining of NP tissues and decellularized tissues (dECM). (C) Representative scanning electron microscopy (SEM) images of the F127, F127 with 0.1% or 1% dECM, and dECM.


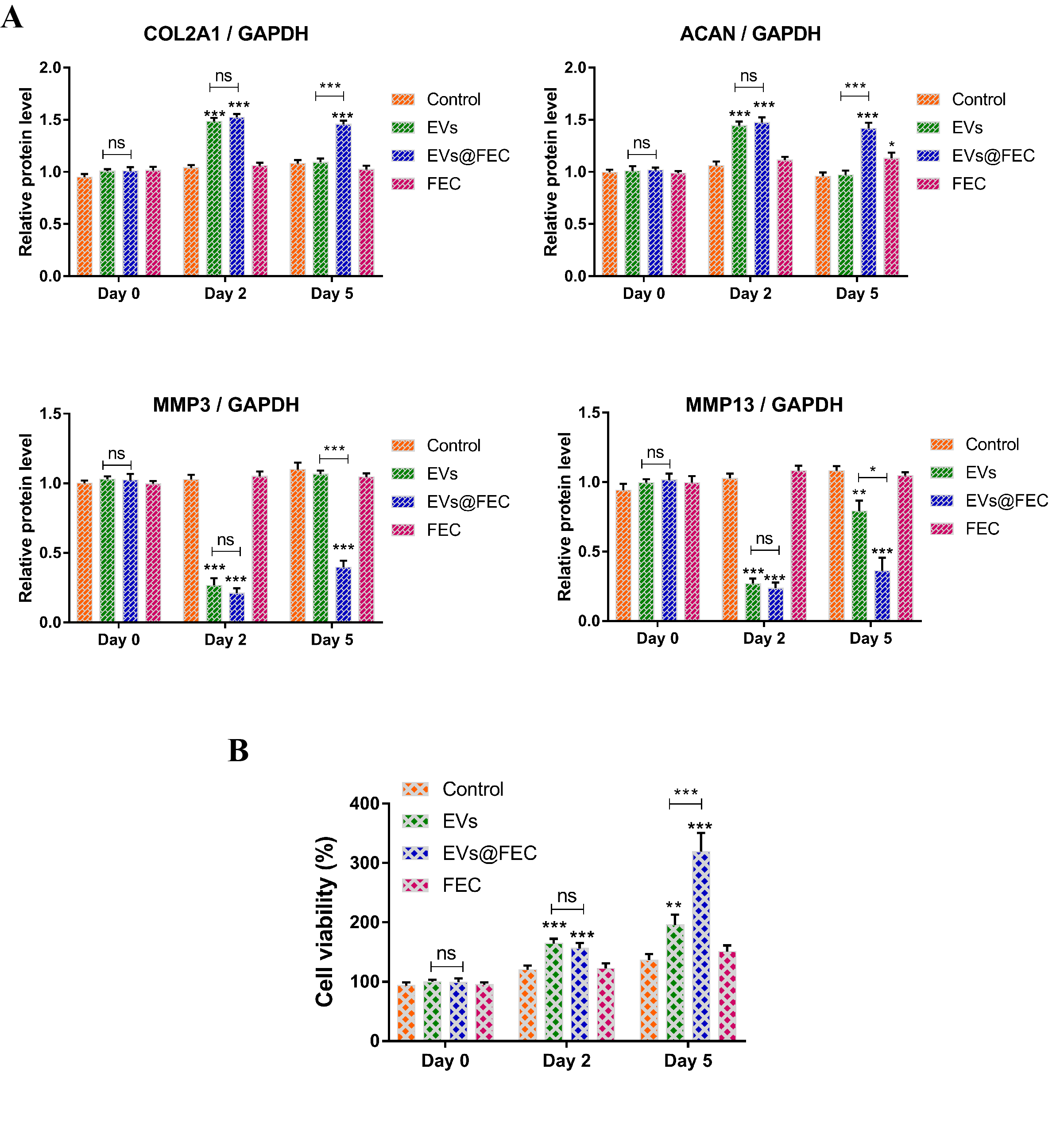


**Figure S4:** NP cells were cultured with EVs, EVs@FEC or FEC at specific time points. (A) The quantification levels of ACAN, COL2A1, MMP3, and MMP13 measured by western blot. (B) NP cell viability was evaluated by CCK-8 assay accordingly. Data were presented as mean ± SD of three independent replicates. P > 0.05 (ns, not significant), *P < 0.05, **P < 0.01, and ***P < 0.001.

**Supplemental tables：**

| **Table S1：Primers of targeted genes** | | |
| --- | --- | --- |
| Name | Forward | Reverse |
| COL2A1 | AGAACTGGTGGAGCAGCAAGA | AGCAGGCGTAGGAAGGTCAT |
| ACAN | TGAGCGGCAGCACTTTGAC | TGAGTACAGGAGGCTTGAGG |
| MMP3 | TTCCTTGGATTGGAGGTGAC | AGCCTGGAGAATGTGAGTGG |
| MMP13 | CCCAACCCTAAACATCCAA | AAACAGCTCCGCATCAACC |
| GAPDH | TCAAGAAGGTGGTGAAGCAGG | TCAAAGGTGGAGGAGTGGGT |

| **Table S2: Sequences of siRNAs** | | |
| --- | --- | --- |
| Name | Sequence (5’→3’) | |
| si-VASN: #1 | guide | ACGUACUUUAGUGGUUACUCdTdT |
|  | passenger | UGCAUGAAAUCACCAAUGAGdTdT |
| si-VASN: #2 | guide | UUCUACGAAAUCCUUGUACAdTdT |
|  | passenger | AAGAUGCUUUAGGAACAUGUdTdT |
| si-VASN: #3 | guide | AACAUUCUGUUUGCUACUAUdTdT |
|  | passenger | UUGUAAGACAAACGAUGAUAdTdT |
| si-NOTCH1: #1 | guide | CUCCUUUAUAGCUGCUAACAGdTdT |
|  | passenger | GAGGAAAUAUCGACGAUUGUCdTdT |
| si-NOTCH1: #2 | guide | CACUGUAGUUGUUGUUACUCAdTdT |
|  | passenger | GUGACAUCAACAACAAUGAGUdTdT |
| si-NOTCH1: #3 | guide | CGGAGUUGUAGGAUGUUCUAGdTdT |
|  | passenger | GCCUCAACAUCCUACAAGAUCdTdT |
| si-scr | guide | UUCUCCGAACGUGUCACGUdTdT |
|  | passenger | AAGAGGCUUGCACAGUGCAdTdT |
